# A public e-survey to explore community understanding of the role of the midwife in Australia

**DOI:** 10.18332/ejm/114226

**Published:** 2019-11-29

**Authors:** Lois McKellar, Angela Brown, Pamela Adelson

**Affiliations:** 1 School of Nursing and Midwifery, University of South Australia, Adelaide, Australia; 2 Rosemary Bryant AO Research Centre, University of South Australia, Adelaide, Australia

**Keywords:** midwifery, midwife, public awareness, continuity of care, model of care

## Abstract

**INTRODUCTION:**

There is compelling evidence that when a woman sees the same midwife there are better outcomes. Yet in Australia, access to midwifery continuity of care remains limited. There are a number of reasons for this but one barrier appears to be a lack of public understanding regarding the role of the midwife. This study undertook an e-survey to explore Australian public perceptions of the role of the midwife.

**METHODS:**

A public opinion sample e-survey, using an exploratory design, a Likert scale and open-ended questions, was distributed through social media over six weeks. The survey was open to Australian residents and was completed by 1657 participants. Of these, 96.9% identified as female and 82.1% of participants had children.

**RESULTS:**

Nearly half of the participants believed that a woman must see a doctor during pregnancy and after birth, compared to 21.9% during birth. Many participants described midwives as caring and supportive but there was a lack of understanding about their level of skill and expertise. A dominant theme was the alignment of medical care with safety and the perception that medical practitioners reduce risk. These misperceptions may impact on women making an informed choice regarding midwifery model of care in Australia.

**CONCLUSIONS:**

There is an underlying public narrative whereby the public primarily associate midwives with birth and perceive them as assistants rather than lead care providers. The study findings informed a public awareness campaign in South Australia conducted to educate the public’s understanding of the role of the midwife.

## INTRODUCTION

There is compelling evidence that when a woman sees the same midwife throughout her pregnancy, birth and following birth, that there are better outcomes for the woman^1-4^. In a recent Cochrane Review, it was found that women who engaged in midwifery continuity of care were less likely to experience interventions, have a lower risk of pre-term birth and more likely to be satisfied with their care^1^. Yet in Australia, access to and provision of midwifery continuity of care remains limited^5^.

In 2010, the Australian National Maternity Services Plan (NMSP) was developed that identified a number of priorities^6^ including recommended changes to improve choice and availability of a range of models of maternity care. These included an expanded role for midwives^6^ who could establish ‘private’ practices, prescribe and access government benefits payments (Medicare Benefits Schedule) covering antenatal services, birth in hospital or birth centre, and postnatal services up to six weeks^7^. Nevertheless, in 2018, the majority of Australia’s maternity care was provided in public and private hospitals, rather than primary care settings, and through medically led services despite the recommendation for an increase in a range of models of care and the known benefits of midwifery continuity of care. In 2016, 97.9% (n=302463) of women gave birth in hospitals, in a conventional labour ward^8^. Additionally, public hospitals provide a significant proportion of antenatal care (55%), as do private obstetricians (30%), while general practitioners (GPs) provide 15% of care^9^.

Many Australian women are seeking, but have limited access to, other models of care such as birthing centres and antenatal/postnatal care in community settings. While a national survey found that 31% of hospitals now offer midwifery caseload care, which includes midwifery group practice and team midwifery models of care, the authors estimated that only 8% of women actually received or had access to caseload care despite high demand for these services^10^. In South Australia, approximately 6% of women access care from a known midwife during pregnancy, birth and after the baby is born, through midwifery group practice and team midwifery models offered through public hospitals^8^.

An option for midwifery care outside of the hospital system is care from a privately practising midwife. However, uptake of this option has been variable and midwives have experienced challenges in setting up and sustaining private midwifery practices, including access to insurance and the development of collaborative relationships with hospitals^11^. Anecdotally, there also seems to be a reluctance from women to engage in private midwifery services outside of conventional hospital settings due to a lack of understanding regarding the full scope and role of the midwife. This potential lack of understanding impacts on the capacity for some women to make an informed choice regarding care options and may have hindered the uptake of midwifery models of care in Australia.

The aims of this study were to explore and describe the public perception regarding the role of the midwife in Australia and inform the development of a public awareness campaign in South Australia to promote an accurate understanding of the role of the midwife. The key research questions for this project were: 1) ‘What are the Australian public’s understandings and beliefs regarding the role of the midwife in Australia?’, and 2) ‘What key messages are needed to inform the public regarding the contemporary role of the midwife in Australia?’.

## METHODS

A public opinion sample e-survey, as described by Brooker and Schaefer^12^, was undertaken in 2017. The methodology involves asking a sample of people their opinions about the issues being considered^12^, with a recommended sample of 400–3000 participants. It is acknowledged that this representation is only as accurate in as much as the people who are surveyed represent the larger population^12^.

The survey was developed by the researchers based on reviewing available literature and was designed as an exploratory questionnaire consisting of a series of ‘yes/no’ and Likert-scale questions to explore the understanding of the role of a midwife. Demographic data were limited to age, gender, and parity. For selected questions, participants were asked to elaborate through open-ended questions. Additional questions were directed at those who had children in order to explore their experiences with previous maternity care providers. For those who had children, the type of provider for the majority of their care was sought and included the options of: GP (alone or shared care), midwife only, public hospital medically-led clinic care, public hospital doctor and midwife clinic, private obstetric care, private obstetric with midwife care, and other. These categories were reduced to two care groups for analysis: i) a medical model of care, described as receiving care from a GP, obstetrician or where there was a combination of care from a doctor and midwife; and ii) a midwifery model of care, described as ‘receiving the majority of care from a midwife’.

An advisory committee was established including midwives and consumer representatives to provide input in the development of the survey questions. The draft survey was provided to the committee to test face validity. Subsequently, changes to wording and organisation of the survey were made to the final version. The online questionnaire was distributed through Survey Monkey^®^.

### Participants

The survey was open to Australian residents who accessed social media for six weeks in the period October–November 2017. It was distributed through Facebook and Twitter with the intention of capturing diversity and representing the Australian general population. A Facebook information page was constructed and included a link to the e-survey that could be further shared through social media for snowball sampling. Midwives, consumers, family and friends were asked to share the Facebook page with the expectation that this would travel broadly and incorporate a wide number of people from a variety of demographic locations and socioeconomic contexts.

Research utilising social media as a recruitment strategy must consider ethical principles such as consent, privacy and anonymity. Before starting the survey, respondents were provided written information about the purpose of the study and were advised that they were free to participate or not. They were also advised that their individual responses would be anonymous and would be used to inform a public awareness campaign. The Facebook post had a link to the survey hosted on an external site (Survey Monkey) that ensured that IP addresses could not be traced. The survey was anonymous, submission of the completed survey was taken to denote consent. To complete the e-survey participants had to be aged ≥18 years and living in Australia. Ethics approval was granted from the University of South Australia Human Research Ethics Committee (No. 200553).

### Analysis

Quantitative data were analysed descriptively using Excel and STATA v14.0. Free text qualitative data were analysed by thematic analysis using open coding, drawing on the Braun and Clarke^13^ framework. Transcripts were read and re-read by the authors and initial codes generated, and grouped into themes through the software NVivo 11. The themes were reviewed and defined through an iterative process undertaken by two of the authors.

## RESULTS

There were 1657 eligible surveys completed in full or in part. Respondents were primarily female (96.9%) and 82.1% of participants had children. Denominators varied according to whether the question was directed at those who had children or all participants, and whether the question was answered. Participants were generally young, in the following age groups: 43.3% (n=718) 26–35 years; 24.1% (n= 399) 36–45 years; 11.5% (n=191) 18–25 years; and 21.1% (n=359) >45 years.

Of those who had had children, 58.1% (n=790) received care in their previous pregnancy through a medical model and 41.7% (n=657) received care through a midwifery model. When asked why they ‘chose’ this type of care over the other model, the overall largest response category was due to their own research, followed by recommendations from family and friends, having private health insurance, and referred by a GP (Table 1). Responses varied depending on the model of care received. Notably, women who researched providers were more likely to choose a midwifery model of care (50.8% midwifery vs 14.1% medical), whereas the combination of lack of knowledge and not aware of choice, were more highly reported with receiving a medical model of care (28.3% medical vs 5.8% midwifery). Additionally, recommendations from family and friends were more likely to result in receiving care through a midwifery model (23.5% midwifery, 12.0% medical). Those who received care through a medical model were more likely to select private health insurance (30.5%) and referral by a GP (23.8%) as the reason for their choice.

**Table 1:** Responses from women who had children to the question ‘Why did you choose this model of care?’

*Reason(s) for choosing medical care or midwifery care (more than one response)*	*Main model of care last pregnancy*					
	*Medical care (n=790)*		*Midwifery care (n=567)*		*Total responses (N=1367)*	
	*n*	*%*	*n*	*%*	*n*	*%*
Reading/researching about providers	111	14.1	288	50.8	399	29.4
Friend or family recommendation	95	12.0	133	23.5	228	16.8
Private healthcare	241	30.5	3	0.5	244	17.9
Referred by GP	188	23.8	57	10.1	245	18.1
Only type of care I/we knew about	144	18.2	19	3.3	163	12.0
Did not realise I/we had a choice	80	10.1	14	2.5	94	6.9
My partner’s preference	22	2.8	17	3.0	39	2.9
Social media	1	0.1	3	0.5	4	0.3
Television (TV)/magazine	0	0	3	0.5	3	0.2
Other reasons (free text)						
My choice	71	9.0	128	22.6	199	14.7
High-risk pregnancy	77	9.8	0	0	77	5.7
Previous experience	22	2.8	40	7.0	62	4.6
Access (no or limited access to care options)	44	5.6	4	0.7	48	3.5
No choice of care or financial considerations	36	4.6	13	2.3	49	3.6
Working as a nurse or midwife	4	0.5	13	2.3	17	1.3
Seeking continuity of care	11	1.4	16	2.9	27	2.0

*Two participants had ‘free birthed’ and two had engaged a doula to birth.

Participants were questioned as to whether they had been given the option of having a midwife to provide the majority of their care throughout pregnancy, birth and postnatally. Approximately equal proportions reported they had (50.4%, n=684) or had not (49.6%, n=672) been given the option to choose a midwife. Of those who had received the majority of care from a midwife, 81.3% (n=461) were given an option to choose this type of care. Of those who had mostly medical care, 71.7% (n=566) were not given the option to choose a midwife for their care. This was analysed further based on type of medical care; those who had private obstetric care indicating they were largely not asked (90.6%) and 77.4% of those who received mostly GP care were not given a choice regarding midwifery care. The majority (60.8%) of those who received care with obstetricians and midwives through the public clinics were also not asked if they wanted to receive a midwifery model of care.

Participants who had children were asked if they would choose a midwife to provide the majority of care in a subsequent pregnancy (Table 2). Of those that had midwifery care, the overwhelming majority (98.1%) responded that they were extremely or very likely to have this type of care again. Of those that had medical care for their previous maternity care, the majority (62.3%) said they were also extremely likely or very likely to choose a midwife to provide the majority of care during the next pregnancy, birth and after birth.

**Table 2:** Responses from women who had children to the question ‘Would you chose a midwife to provide the majority of care for your next pregnancy, birth and following birth?’

*How likely to choose a midwife in future care*	*Main model of care last pregnancy*			
	*Medical model*		*Midwifery model*	
	*n*	*%*	*n*	*%*
Extremely likely	335	45.0	520	94.6
Very likely	129	17.3	19	3.5
Moderately likely	152	20.4	8	1.5
Not very likely	80	10.8	1	0.2
Not at all likely	48	6.5	2	0.4
Total	744	100	550	100

In a series of ‘yes/no’ questions all participants were asked whether women must see a doctor during pregnancy, labour, birth and after the baby is born (Table 3). Nearly half of the participants believed that a woman must see a doctor during pregnancy (48.2%) and after the baby is born (44.7%). Interestingly, less than one-quarter believed that women must see a doctor during birth (21.8%).

**Table 3:** Responses from women who had or did not have children, when asked ‘When do you think a woman must see a doctor?’

*When do you think a woman must see a doctor?*	*Participants who had children*				*Participants who did not have children*				*Combined participants’ responses*			
	*No*		*Yes*		*No*		*Yes*		*No*		*Yes*	
	*n*	*%*	*n*	*%*	*n*	*%*	*n*	*%*	*n*	*%*	*n*	*%*
During pregnancy	688	52.8	614	47.2	140	47.6	154	52.4	828	51.8	769	48.2
During labour	1058	81.5	240	18.5	222	75.2	73	24.8	1280	80.3	314	19.7
During birth	1035	79.8	262	20.2	210	71.2	85	28.8	1245	78.2	348	21.8
After the baby is born	740	56.9	560	43.1	143	48.5	152	51.5	883	55.3	713	44.7

All participants were asked if they knew how to find a midwife to provide the majority of their care. Overall, approximately one-third of participants (32.4%) identified they would not know how to find a midwife for their care. This was influenced by child status, with approximately half (45.1%) of participants who did not have a child not knowing how to find a midwife, whereas only 29.5% of women who already had a child were unaware how to find a midwife. Overall, when participants were asked what would be the best way to find information about receiving care from a midwife, 61.3% of participants listed GP as the best source for finding a midwife, followed by Facebook (23.1%) (Table 4).

**Table 4:** Responses* from participants who had or did not have children, when asked ‘How would it be best for you to find information about receiving care from a midwife?’

*Response categories*	*Participants who had children*		*Participants who did not have children*		*Total*	
	*n*	*%*	*n*	*%*	*n*	*%*
Facebook	274	22.7	69	24.9	343	23.1
General Practitioner	753	62.3	158	57.0	911	61.3
Television	141	11.7	41	14.8	182	12.2
Billboard advertising	13	1.1	4	1.4	17	1.1
News or magazine	28	2.3	5	1.8	33	2.2

* N=253 additional free text responses, most common response: internet/Google search/specific websites and all the above categories.

The survey asked participants to provide three words or phrases that came to mind when they thought of a midwife and 98.8% (n=1636) provided descriptors. Responses were positive with only seven negative comments referring to midwives engaging in ‘dangerous’ homebirths, over-emphasising breastfeeding, giving conflicting advice, or they were judgemental.

**Figure 1:**
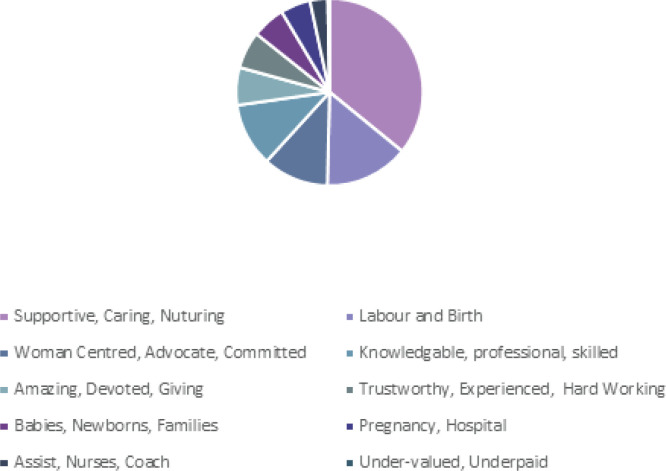
Responses from respondents who have children, when asked ‘What three words or phrases come to mind when you think of a midwife?’

**Figure 2:**
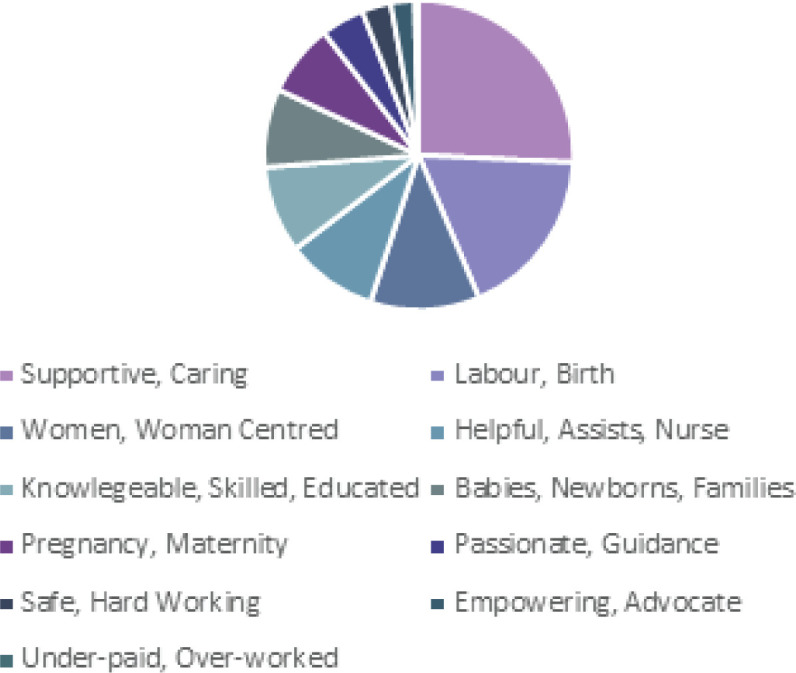
Responses from respondents who did not have children, when asked ‘What three words or phrases come to mind when you think of a midwife?’

Overwhelmingly, the most common words to describe a midwife by all participants were ‘supportive and caring’, with additional words such as ‘helpful, kind, nurturing and devoted’ used repeatedly. This was followed by words related to labour and birth and being knowledgeable, professional, and woman centred. The responses of those who had children varied in some aspects from the responses of those who had not had children (Figures 1 and 2). Those who had not had children described midwives more often as assistants than as knowledgeable. Those who had experienced having a baby also used words related to being safe and trustworthy considerably more often than those who had not, however for both groups of participants this was less than other descriptors.

**Figure 3:**
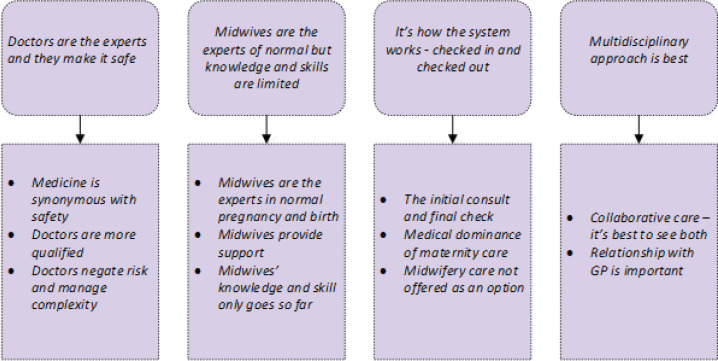
Thematic analysis of reasons given for why women should see a doctor during pregnancy, birth and following birth

The participants who indicated that a woman must see a doctor during her maternity care were asked to provide a rationale for their response. In total, 889 participants responded to this question: 720 (81%) who had a child and 169 (19%) that did not. Four themes were identified, each with a number of subthemes (Figure 3). A summary and examples of comments from the four themes and subthemes follows.

### Theme 1: Doctors are the experts and they make it safe

Nearly half of all participants who responded indicated that they should see a doctor during pregnancy and after birth. This appears to be linked to a belief that a medical practitioner’s expertise could make maternity care safer as described in the sub-themes.

#### Medicine is synonymous with safety

‘I believe during and after pregnancy it is important to see a doctor to ensure you and your baby are safe and everything is moving in the right direction, I feel safer with a qualified doctor to give me the ok that everything is fine’. (P1483)

There was also a sense that the doctor should be there ‘just in case’:

‘See a doctor also through, during and after birth just in case there maybe something that the midwife can’t do or answer’. (P1611)

Some participants believed that a doctor was needed to ‘oversee’ the midwife’s care and that this made it safer:

‘A doctor should be present at times to ensure everything is well, no medical interventions are required, ensure the midwife is giving appropriate care to the mother and baby’. (P1298)

#### Doctors are more qualified

This sub-theme identified where participants thought that medical practitioners were more qualified than midwives:

‘It is true that doctors are more qualified than midwives so I find it important for a doctor to see these stages to ensure that everything is working normal at these stages for the safe delivery of the child and safety of the mother’. (P1340)

Some participants described doctors as having a larger scope of practice that contributed to their expertise:

‘From my understanding, doctors can diagnose where midwives can’t. So, if I had a question about something not “normal” to pregnancy, I assume doctors can give a more detailed answer or diagnose’. (P1145)

There also seemed to be assent to the authority of the medical practitioner for these participants:

‘I value the care that midwives provide but I prefer doctors to have the final say’. (P1548)

‘Doctors are in charge of making decisions for pregnant women and babies and providing more specific care’. (P1654)

Alongside the belief that doctors were more qualified was a misunderstanding that doctors were required for a variety of skills that midwives did not have, such as:

‘Pregnancy: initial confirmation of pregnancy, booking of ultrasound, blood tests. Birth: in my case, if stitches are required for any tearing. After baby is born: to do routine checks, such as hips, hearing, general health of baby etc.’ (P1349)

#### Doctors negate risk and manage complexity

The comments indicated that there was a concern about risk that could be mitigated with the involvement of a doctor:

‘They don’t have to see a doctor, but it’s best practice if we want to reduce complications and risks’. (P767)

‘I feel you need the medical expertise and be under the supervision of a doctor through each stage of pregnancy and birth to ensure safe outcomes for mother and baby and lower any risk’. (P1504)

There was also commentary on the responsibility of doctors to manage risk and complexity if these arose during pregnancy, birth or after the baby was born:

‘If there were serious medical complications, I would expect that a doctor would be the one to provide the appropriate medical care’. (P1370)

With a focus on risk, there was also an underlying narrative that midwifery care could be risky:

‘Because although midwives technically can do all the things listed, their proficiency at them is hit and miss. This is not what you want for a birth situation. Although some are obviously great, the overall picture is a confusing, conflicting’. (P1443)

‘Midwives get it wrong’. (P1598)

### Theme 2: Midwives the experts of normal birth but knowledge and skills are limited

Midwives were recognised by some participants as experts in normal birth but there was a belief that their knowledge and skills were limited.

#### Midwives are the experts in normal pregnancy and birth

‘Midwives are incredibly knowledgeable and instinctive during labour & birth, in my experience with three straight forward labour/births, the midwives always knew what was going on and what decision to make’. (P854)

‘I believe midwives are birth specialists’. (P1159)

#### Midwives provide support

Participants saw midwives strongly positioned in providing support for women:

‘But my midwife gave me the skills to cope, courage to move forward’. (P1430)

‘I used a midwife for the majority of my care during my second pregnancy and couldn't be happier with the way it all went. I always felt safe and cared for throughout pregnancy, during the birth and after the baby was born’. (P964)

#### Midwives’ knowledge and skills only go so far

There was a clear lack of understanding regarding the midwives’ level of knowledge and skills. Many participants stated that the midwives’ knowledge and skills only went ‘so far’ and a doctor was required to fill the gap:

‘Whilst midwives are well educated in their chosen field, they are not doctors, sometimes advice from doctors is necessary’. (P1646)

‘I am unaware if a midwife is qualified to be the only medical personnel to handle a new pregnancy and all that entails’. (P948)

### Theme 3: It’s how the system works - checked in and checked out

The responses described a system that the public engaged in to receive maternity care, whereby women needed to be ‘checked in and checked out’.

#### The initial consultation and final check

Participants believe the system requires women to see a doctor for pregnancy confirmation and to book into maternity care and also to see a doctor either before leaving hospital or at six weeks after the baby is born to be formally discharged. Comments revealed that most participants believed a doctor was necessary for pathology, scans, prescriptions, contraception, and immunisation:

‘I think usually people initially need to see a GP to confirm their pregnancy, arrange blood tests and discuss referral to an obstetrician/midwife/hospital/birthing centre. After birth I think it is important for the baby to be checked over by a paediatrician’. (P1199)

‘I always thought that women had to see a doctor during pregnancy, because that is what was always done. I also thought that the Dr had to discharge the woman and baby after the Dr had checked them both after delivery’. (P1152)

#### Medical dominance of maternity care

Many participants believed that medicine was the dominant narrative in maternity care:

‘My GP was very reluctant to allow me to only see a private midwife for my care I had to fight quite hard just for a referral’. (P711)

‘I think that is the way the system is set up - you have to really try for midwife only care’. (P1536)

#### Midwifery care not offered as an option

There were frequent comments indicating participants did not know that midwifery care was an option for their care and that it was not offered to them as a maternity care choice:

‘I didn't know that was an option to not have a doctor involved’. (P1494)

‘Was always told to see both, I could always see the midwife more but had to see my doctor occasionally. Would have rathered (sic) just seen my midwife, as my doctor wasn't the one that delivered my baby anyway and my midwife was amazing’. (P887)

‘I don’t think first time mothers like myself know enough about midwives & their expertise. GPs need to let women know all their options’. (P1164)

### Theme 4: Multidisciplinary approach is best

Participants also commented about the need for a multidisciplinary approach and that collaboration could be a positive aspect of care.

#### Collaborative care – it’s best to see both

Some participants believed that seeing both a doctor and a midwife was better and understood this as a team approach to managing complexities. Whereas others felt this was just a better approach than seeing either a doctor or midwife:

‘There's a combination of midwives and doctors needed in all aspects of pregnancy, labour, postnatal. They provide complimentary but different roles’. (P1625)

‘I think that the ideal pregnancy/birth is where both doctors and midwives are involved and work together’. (P1538)

#### Relationship with the GP is important

Some participants indicated that keeping a relationship with the GP was important for continuity of care to the family and a good idea as they would provide ongoing care as the baby grew:

‘I think it is important as part of ongoing medical care that women see their GP during their pregnancies and after the baby is born’. (P1185)

‘It is important to involve a GP in prenatal and postnatal advice, as this may impact future care’. (P1227)

## DISCUSSION

The findings indicate that confusion exists for the public regarding the role and scope of the midwife in Australia. In particular, that midwives can provide care from conception through to six weeks after the baby is born, not simply during birth. The public holds midwives in high regard and describe midwives as supportive, caring and woman centred, but they predominantly associate midwives with labour and birth. It was also evident that many perceive midwives as assistants within maternity care services rather than lead maternity care providers.

A dominant theme in this study was the alignment of medical care with safety. It has been suggested that a discourse of fear, risk and safety is embedded in everyday birth language creating a narrative that affirms medicalised care^14,15^. It was clear that ‘risk’ and ‘safety’ were concerns for the public and that medical practitioners were perceived to reduce risk and increase safety. Alongside this, there was a lack of knowledge regarding the level of education of midwives and the extent of their clinical skills. The participants seemed unsure of the midwives’ expertise, whereas they described the medical practitioners as being highly qualified experts. This focus on risk and safety, as well as lack of evidence-based information has been identified in a number of studies as a key predictor for choices women made^14,16,17^.

A study in the UK that explored the influences on the place of birth for women, found that concern for safety was the main influence for birth in hospital with participants acknowledging that being able to access medical staff readily contributed to a sense of safety^16^. The authors concluded that beliefs about risk and safety ultimately impacted on decision making about place of birth. In a review, Hadjigeorgiou et al.^17^ found that much of the literature suggested that women saw birth as risky with the potential for complications at any time. This perception of safety shaped a woman’s choice for the model of care. Interestingly, where the woman felt confident in the midwife’s knowledge and expertise, they were more likely to seek birth with a midwife at home^17^.

Gaining information about options for birth is not always easy. In this study, a third of participants did not know how to find a midwifery care option and the majority of participants who had received care through a medical model had not been provided information or offered a choice about midwifery care options. Similarly, women in the study by Hadjigeorgiou et al.^17^ indicated that health professionals, even midwives, did not always freely provide information on options of care. The media may contribute to this lack of knowledge, as examples of normal birth with midwifery care are lacking and birth is more commonly portrayed as being complex and incorporating the need for medical intervention^18^. In a critical analysis of articles, published in The Age (Australian Newspaper), McIntyre et al.^19^ concluded that while the media continues to perpetuate a message of medical dominance, a new narrative is being provided. One that recognises that Australian maternity services are inflexible and outdated but this is coupled with the notion that change could undermine what is an inherently safe system of care^19^. This creates conflict and confusion for the general public, about the evidence, which is still unsure about care through midwifery models.

With the notion of medicalised care being clinically safe, not simply in the beliefs of women but also in those of health professionals, the medical model remains a powerful influence on maternity care^17^. According to Fahy and Parratt^20^, childbirth within the Australian health system is still considered by many as a medical process that should be managed in hospital. This belief in the medical model was apparent throughout this study, as participants described a need to be ‘checked in’ by a medical practitioner to engage in the system and also to be ‘checked out’. Additionally, there was a strong theme throughout the participants’ comments that identified medical practitioners as the experts in maternity care. This is not surprising as this understanding is grounded in history and has been perpetuated in modern discourse^21,22^. Significantly, this lack of understanding may impact on a woman’s choice of provider and access to care from a known midwife. When the participants indicated that they had undertaken their own research on available models of care, they were more likely to have had the majority of care from a midwife. Additionally, recommendations from family and friends influenced the choice for midwifery care, whereas private health insurance and referral by a general practitioner were more likely to result in a medical care option. These findings are consistent with previous research^23^ that found that choice for pregnant women seems to be primarily determined by three factors: the knowledge women possess about care models, local availability of services and perceptions of risk, acknowledging that sociodemographic factors may also play a part.

Overall, the knowledge Australian women possess about pregnancy care seems to be highly variable and sources of information may influence their choice. Providing GPs with the evidence supporting midwifery continuity of care models, as well as explaining the role and scope of the midwife, may be beneficial.

The public perceive midwives positively, however, there is a need to re-educate the public and health professionals about the role of the midwife^24^. In this study, 98% of women who experienced care through a midwifery model indicated they would choose this option again, suggesting high satisfaction as reported in the most recent Cochrane review on midwifery continuity of care^1^. This growing body of evidence positions midwives to confidently continue to advocate for evidence-based midwifery continuity of care for women and their families.

In response to the lack of understanding about midwifery within the wider community, a public awareness campaign was developed for South Australia. This campaign included transit posters on public buses, radio interviews and a social media strategy to share information and a series of intentionally designed videos, all informed by the study findings. A website was constructed to provide the public with a platform to gain evidence-based information about the benefits of care from a known midwife and a contact point to provide women with further information and how to access midwifery services. Specifically, the campaign responded to the findings of this study by showcasing the midwife as a caring health professional who provides safe care through a relationship-based approach, placing women and their families at the centre of care. The concept of women and midwives being together on this journey is captured in the phrase “we’re in this together”, which has been used consistently throughout the resources. To address the misunderstanding regarding the scope and breadth of midwifery care, the campaign focussed on the provision of care during pregnancy and after the baby is born, depicting this in both hospital and home environments. Midwifery students have been included in the campaign to show the public that being a midwife requires extensive academic study and significant practice-based experience with mentoring by practicing midwives. The campaign highlighted the fact that midwives are highly educated and highly skilled health professionals. The campaign deliberately included collaboration as a key aspect of how midwives work in order to reassure the public that midwifery care is safe and that midwives work together with other health professionals as needed, in particular with the medical profession when complications arise. Campaign videos can be accessed from https://www.midwives.org.au/your-journey-known-midwife.

### Limitations

One of the limitations using the internet as a means of collecting data is a lack of control over who responds and who does not, therefore it is not clear if non-participants differ in demographics and opinion from participants^25^. As such, the non-probability sampling limits the generalisability of the results, however this was not a primary aim of this survey. A further limitation in this study is that the electronic medium used to share the survey was predominantly Facebook. To commence data collection, colleagues, midwives, family and friends were asked to share the study Facebook page and survey link. There is potential that this distribution was not representative of the population, as this group of early participants may have had a greater understanding regarding the role of the midwife. This was possibly evident in the larger than anticipated number of participants who stated that they had received the majority of their care from a midwife. It is also possible that in describing midwifery care as receiving the ‘majority of care from a midwife’ that this may have been interpreted inconsistently. For example, this may have included hospital care whereby care was predominantly provided by a midwife in a clinic. It was challenging to formulate a descriptor to capture a midwifery model of care that the general public would understand.

## CONCLUSIONS

This research sought to understand the public perception of the role of the midwife in Australia and was used specifically to inform a public awareness campaign. While midwives are viewed positively by the public, there is a need for ongoing and increased momentum to showcase midwives as lead maternity care providers who are safe and work collaboratively, and to promote the public health impact of their role.
